# Excitotoxic lesions of the perirhinal cortex leave intact rats’ gustatory sensory preconditioning

**DOI:** 10.1177/17470218211054981

**Published:** 2021-11-02

**Authors:** Jasper Robinson, Peter M. Jones, Emma J. Whitt

**Affiliations:** 1 School of Psychology, University of Nottingham, Nottingham, UK; 2 School of Psychology, University of Plymouth, Plymouth, UK

**Keywords:** Sensory preconditioning, stimulus representation, associative learning, perirhinal cortex

## Abstract

We report findings from two sensory preconditioning experiments in which rats consumed two flavoured solutions, each with two gustatory components (AX and BY), composed of sweet, bitter, salt, and acid elements. After this pre-exposure, rats were conditioned to X by pairing with lithium chloride. Standard sensory preconditioning was observed: Consumption of flavour A was less than that of B. We found that sensory preconditioning was maintained when X was added to A and B. Both experiments included one group of rats with lesions of the perirhinal cortex, which did not influence sensory preconditioning. We discuss our findings in the light of other sensory preconditioning procedures that involve the perirhinal cortex and conclude that differences in experimental variables invoke different mechanisms of sensory preconditioning, which vary in their requirement of the perirhinal cortex.

Sensory preconditioning (e.g., Dwyer et al., 2012; Prewitt, 1967; Rescorla, 1983; Ward-Robinson et al., 1998; Westbrook et al., 1995) has proven to be instructive in the study of learning about the co-occurrence of neutral events. A typical experiment involves three stages. In the first stage, pre-exposure, stage subjects are presented with a pair of relatively neutral stimuli (A and X; such as a tone and a light or a solution that is both bitter and salty). In the following conditioning stage, stimulus X serves as a cue for an unconditioned stimulus (+), establishing the conditioned response. The crucial test involves the presentation of stimulus A, which will elicit the conditioned response established to stimulus X. Because A has never been paired with +, the conditioned responding it elicits is only possible if the initial pairing of A and X resulted in learning about their co-occurrence.

Holmes et al. (2013; see also, Holmes et al., 2018; Nicholson & Freeman, 2000) demonstrated the importance of the perirhinal cortex in sensory preconditioning. The perirhinal cortex comprises Areas 35 and 36 and, in the rat, is located in the rostral and middle portions of the rhinal sulcus (see, for example, Burwell, 2001). In Holmes et al.’s procedure, rats received an audio-visual, serial compound stimulus, A → X before X was established as a conditioned stimulus for a foot-shock unconditioned stimulus. The conditioned response (visually assessed freezing) was seen, after the two training stages, to stimulus A in control rats. This sensory preconditioning effect was markedly reduced in a second group of rats whose perirhinal cortices were temporarily inactivated during the initial A → X pairings. Inactivation was achieved by the cannulated infusion of muscimol and was specific in reducing responding to stimulus A: Responding to X was unaffected, thus eliminating the interpretation that the inactivation rendered learning (generally) ineffective or the shock less effective.

Evidence from anatomy and from behavioural neuroscience concurs with this aspect of Holmes et al.’s (2013) report. The perirhinal cortex is in receipt of input from multiple sensory modalities (Burwell & Amaral, 1998), which could, when not inactivated, support the integration of the auditory and visual stimuli (A → X). Object memory experiments with rats (for a summary, see, for example, Robinson & Bonardi, 2015) have shown the perirhinal cortex to be involved in recognition memory (e.g., Albasser et al., 2009; Liu & Bilkey, 2001; Mumby & Pinel, 1994; Warburton et al., 2003; Winters et al., 2004). The simplest comparator theories of recognition (e.g., Konorski, 1967; Sokolov, 1963; Stein, 1966; Wagner, 1979) require to-be-recognised stimuli to be compared to, and matched with, a stable, internal representation of the stimulus, formed as a result of previous exposure (cf. Fernández & Tendolkar, 2006). Based on a series of analytical experiments, Rescorla (1981; see also, Lin et al., 2017) suggested that sensory preconditioning may also rely on A and X acting as a unitary representation, AX. During conditioning, X’s presentation elicits AX, through its perceptual similarity to X; and on test, stimulus A will, similarly, be mistaken for AX. Here, the learning about the complex, sometimes, multi-modal, stimulus features of the recognised object parallels the learning about the relationship between audio-visual elements of the compound stimulus in Holmes et al.’s report. Thus, the perirhinal cortex may underpin a representational learning process, common to object recognition memory tasks and sensory preconditioning.

However, other explanations besides Rescorla’s (1981) are available to explain sensory preconditioning—the simplest being that test performance is delivered by an associative chain of the form: A → X → + (cf. Wagner, 1981). Here the pre-exposure training results in a standard, albeit behaviourally silent, association between A and X; conditioning establishes the second link in the associative chain between X and +. Stimulus A can, therefore, elicit responding on test without recourse to the idea that A and X’s presentation formed the integrated representation, described above. This is a purely associative solution. Second, Rescorla’s suggested mechanism for AX-mediated sensory preconditioning was based on analytical sensory preconditioning experiments in which A and X’s pre-exposure had *simultaneous* onset and termination. Rescorla maintained (e.g., p. 66) that the AX representation formation was the result of A and X never having been presented separately, meaning that the subject has no reason to perceive them as separate stimuli. Or, to put it another way: Why should the subject perceptually parse the stimuli as A and X (as the experimenter has composed the pre-exposure trial) when it has experienced neither element in the absence of the other? However, rats in Holmes et al. (2013) report received pre-exposure in a *serial*, A → X, compound, which could encourage their being perceived as separate stimuli. Thus, Holmes et al.’s sensory preconditioning could, instead, be the result of the associative chain mechanism—an interpretation supported by Higgins and Rescorla’s (2004) demonstration that extinguished simultaneous associations are more poorly reformed than serial associations.

The current pair of experiments examined the role of the perirhinal cortex in sensory preconditioning in groups of rats with either excitotoxic lesions of the perirhinal cortex or sham lesions. We employed Rescorla and Cunningham’s (1978) sensory preconditioning procedure. Rats are pre-exposed to gustatory stimuli A and X, presented as a simultaneous compound flavour, thus encouraging AX-mediated learning, rather than associative chain learning. We included an additional test to the potential role of the associative chain mechanism of sensory preconditioning. We found evidence of sensory preconditioning which did not solely depend on an associative chain, but unlike Holmes et al. (2013), we found that learning was not dependent on the integrity of the perirhinal cortex.

## Experiment 1

The design of Experiment 1 is summarised in Figure 2. Rats received either excitotoxic lesions of the perirhinal cortex (group PeRh) or sham lesions (group Sham) and, after recovery, underwent the gustatory sensory preconditioning procedure summarised in the top panel of Figure 2. Rats received pre-exposure to flavoured solutions, AX and BY before discriminative conditioning of the form X+, Y−, where + represents emesis created by an injection of lithium chloride. The findings of Holmes et al. (2013) encourage the expectation that the consumption of flavour A would be reduced relative to that of B—the sensory preconditioning effect—in group Sham but that this would be diminished in group PeRh. If, however, our simultaneous means of pre-exposure of A and X determines a qualitatively different form of sensory preconditioning from Holmes et al.’s serial pre-exposure procedure, we might expect equivalent sensory preconditioning in both groups.

### Method

#### Subjects and surgery

In total, 24 male Lister-hooded rats served as subjects. Rats were fully naïve to the stimuli employed in the current experiment and had not before been in a state of water restriction. Rats were experimentally naïve before surgery (see below) but had served in experiments before those reported here. They first served in a conditioned suppression experiment with auditory stimuli and under mild food restriction to encourage instrumental responding for food. After that, they served in two similar procedures involving their placement in arenas in which they encountered innocuous domestic items such as bottles and vases. Rats were not food deprived during these procedures. At least 2-week interval was interpolated between each procedure during which rats were treated as described in the “Apparatus and Stimuli” section during non-experimental periods. Rats’ mean weight before the experiment began was 610 g (range: 525–690 g), and there was no weight difference between groups Sham and PeRh, *t*(15) = 1.20, *p* > .240.

During surgery, each rat was maintained under anaesthesia using a mixture of isoflurane and oxygen. Following establishment of anaesthesia, rats’ scalps were shaved, and rats were placed in a stereotaxic frame (Kopf Instruments, Tujunga, CA) with the incisor bar set to −3.3 mm. An incision was made along the scalp’s midline, which, along with the temporal muscles, was retracted to expose the skull. A small region of skull approximately 3–7 mm posterior to bregma (above each hemisphere’s parietal cortex) was drilled. Ibotenic acid (Sigma Aldrich, Gillingham, UK) was dissolved in phosphate-buffered saline (pH 7.4) to produce a solution of ibotenate of 63 mM. Five microinjections of ibotenate solution, with a 2-µL Hamilton syringe (Hamilton, Bonaduz, Switzerland), were made in each hemisphere at (a) anterior–posterior (AP) = −3.0 mm, medial–lateral (ML)= ±5.8 mm, dorsal–ventral (DV) = −4.0 mm (0.120 µL); (b) AP = −4.0 mm, ML = ±6.1 mm, DV = −3.8 mm (0.100 µL); (c) AP = −5.0 mm, ML = ±6.5 mm, DV = −4.0 mm (0.070 µL); (d) AP = −6.0 mm, ML = ±6.7 mm, DV = −3.5 mm (0.050 µL); (e) AP = −7.0 mm, ML = ±6.3 mm, DV = −3.1 mm (0.035 µL). Here, AP coordinates are with reference to bregma, ML to the midline, and DV to the top of the cortex. The syringe’s plunger was attached to a microdrive (KDS 310, KD Scientific, New Hope, PA), regulating the volume and rate of the ibotenate infusion. Ibotenic acid was delivered at a rate of 0.03 µL/min. Following each infusion, the Hamilton syringe was left in place for 2 min to permit infusion of the solution into the target tissue region. Rats in group Sham received anaesthesia and drilling as described for group PeRh but with no microinjection or needle insertion. Group Sham rats’ duras were perforated with a 25-gauge microlance needle (BD, Ireland). Rats’ assignment to group PeRh or group Sham was random with the constraint that group Sham had 8 rats and group PeRh had 16 rats. For rats in both groups, the scalp incisions were sutured at the end of the procedure, and the rats were placed in a warm recovery box until they exhibited normal behaviour (typically within 24 hr). The recovery box was held in a darkened room illuminated only by red light. Rats in group PeRh received a subcutaneous 5 mL injection of saline and glucose solution. The current procedure commenced 4 months after surgery.

Once the rats had recovered sufficiently, they were transferred back to their home cages, which were held in an air-conditioned vivarium that was illuminated by fluorescent strip lights between 07:00 and 19:00. Temperatures were maintained between 20°C and 23°C. Rats were housed in acrylic cages (20 cm high, 24 cm wide, and 41 cm long) with steel cage ceiling that included a food hopper and a support to hold a water bottle spout. To provide rats with environmental enrichment, home cages contained a large cardboard cylinder, and rats were pair housed and handled daily. Access to fluid was restricted (see “Procedure” section). Home cages contained fresh wood-chip bedding and free access to dry food (Harlan Teklad, Bicester, United Kingdom). Rats were not given supplementary water because we had found in previous experiments that they would exhibit neophobic response to the quinine flavours during the initial pre-exposure trials. However, to promote rats’ consumption of food under water restriction, rats were weighed daily and were offered “wet mash” when weights reduced. This consisted of the rats’ standard food that had been soaked in tap water. Wet mash was presented to rats in a 6-cm diameter ramekin in the rats’ home cages. Rats received daily health checks and remained in good condition throughout the experiment.

After completion of the experiment (see “Procedure” section), the rats in group PeRh were anaesthetised with sodium pentobarbital (200 mg/kg) and intracardially perfused with 9% (w/v) saline. This was followed by a 10% formal saline solution, which was created by adding three parts of 9% (w/v) saline with one part of 40% formaldehyde. The rats’ brains were then removed and postfixed in a 5% formal saline solution. Before sectioning, brains were steeped overnight in 20% sucrose solution. Coronal sections were cut at 40 μm using a freezing microtome. Sections were mounted onto gelatine-coated slides, which were then stained with cresyl violet. Sections were viewed microscopically to determine the extent and location of excitotoxic damage by reference to a brain atlas (Paxinos & Watson, 2005) and with reference to terminology used by Arnault and Roger (1990) and Burwell and Amaral (1998).

#### Apparatus and stimuli

Rats were presented with flavoured tap water (see “Procedure” section) when singly housed in experimental cages, located in the rats’ vivarium. Experimental cages were similar to home cages but did not contain bedding. Tap water was used to make flavoured solutions of 0.33 M sucrose (sweet flavour), 0.16 M sodium chloride (salt flavour), 60.00 M quinine monohydrochloride dihydrate (bitter flavour), and 0.01 M hydrogen chloride (acid flavour). These solutions were presented either alone or mixed, preserving their molarities, and they were administered using inverted 50 mL centrifuge tubes with ball-bearing-tipped spouts. A balance was used to record fluid consumption to the nearest 0.1 g.

#### Procedure

A schedule of water deprivation was established: Water bottles were removed overnight and, on each of the next 2 days, access to fluid was restricted to once-daily water for 1 hr at 11:00. Pre-exposure began on the following day. Over 8 days, all rats received four presentations of each of two compound solutions, AX and BY. For half of the rats, acid was used as X and bitter was used as Y; for the remainder, the role of those flavours was reversed. Within each of these subgroups, for half of the rats, sweet was used as flavour A and salt was used as flavour B; for the remainder of the rats, the role of those flavours was reversed. Rats from groups Sham and PeRh were represented similarly in each of the four counterbalanced subgroups. Solutions were presented in the sequence AX, BY, BY, AX, AX, BY, BY, AX for half of the rats and in the sequence BY, AX, AX, BY, BY, AX, AX, BY for the remainder of the rats. The initial presentations of AX and BY were of only 10 g but this increased on the remaining three presentations of each flavour to 15 g. Solutions were presented for 1 hr at 11:00. Throughout the experiment, the rats’ consumption of the solutions was determined by the difference in the weight of the rats’ tubes before and after presentation.

Conditioning began on the day after the end of pre-exposure and occurred over the course of 5 days. On Days 1 and 4, rats received 15 g of X that was followed, within 20 min of consumption, by an intraperitoneal injection of lithium chloride (0.30 M, 10 mL/kg). Y was presented on Days 2 and 3 but no injection followed. Solutions were presented at 11:00 for 30 min. Rats were also given free access to tap water for 30 min at 15:00. On Day 5, rats received no flavoured stimuli but were offered tap water at 11:00 and 15:00 for 30 min.

On the next day, rats were given 30 min free access to A and B to test for sensory preconditioning. Two tubes, one containing only flavour A and the other containing only flavour B, were presented simultaneously. The tubes’ spouts were 6 cm apart. The solutions A and B were positioned on the left- and right-hand sides equally often across the subgroups.

Standard parametric analyses were used for null hypothesis testing. Tests evaluated two-tailed hypotheses and alpha = .050. Partial eta squared $\left(\eta_{\text{p}}^{\text{2}}\right)$ was used to represent the main effect and interaction effect sizes. Standardised 90% confidence intervals for $\eta_{\text{p}}^{\text{2}}$ were computed using the methods described by Kelley (2007) and used his MBESS package for R (Version 3.3.2 [Computer software], Vienna, Austria). A Bayesian analysis supplemented the interpretation of key results (JASP [Version 0.8 Beta 5] [Computer software], Amsterdam, The Netherlands). The Bayes factor (BF) specifies the ratio of the probabilities between a target model (BF10) and an appropriate comparison, such as the null model (BF01). The magnitude of the ratio is taken to reflect the likelihood of the support for the target model, which may be instructive in interpreting data. Jeffreys (cited in Rouder et al., 2009) maintains that BFs greater than 3 may be considered *some evidence* for one hypothesis over its alternative hypothesis, with BFs of 10 or more or 30 or more as, respectively, *strong* and *very strong* evidence. Unlike standard parametric analyses, Bayesian analyses offer a meaningful interpretation of null results in which the likelihood of the null model can be computed and compared to alternative models. This feature is helpful in studies which rely on the interpretation of a mixture of positive and null results.

### Results and discussion

#### Histology

**Figure 1.**
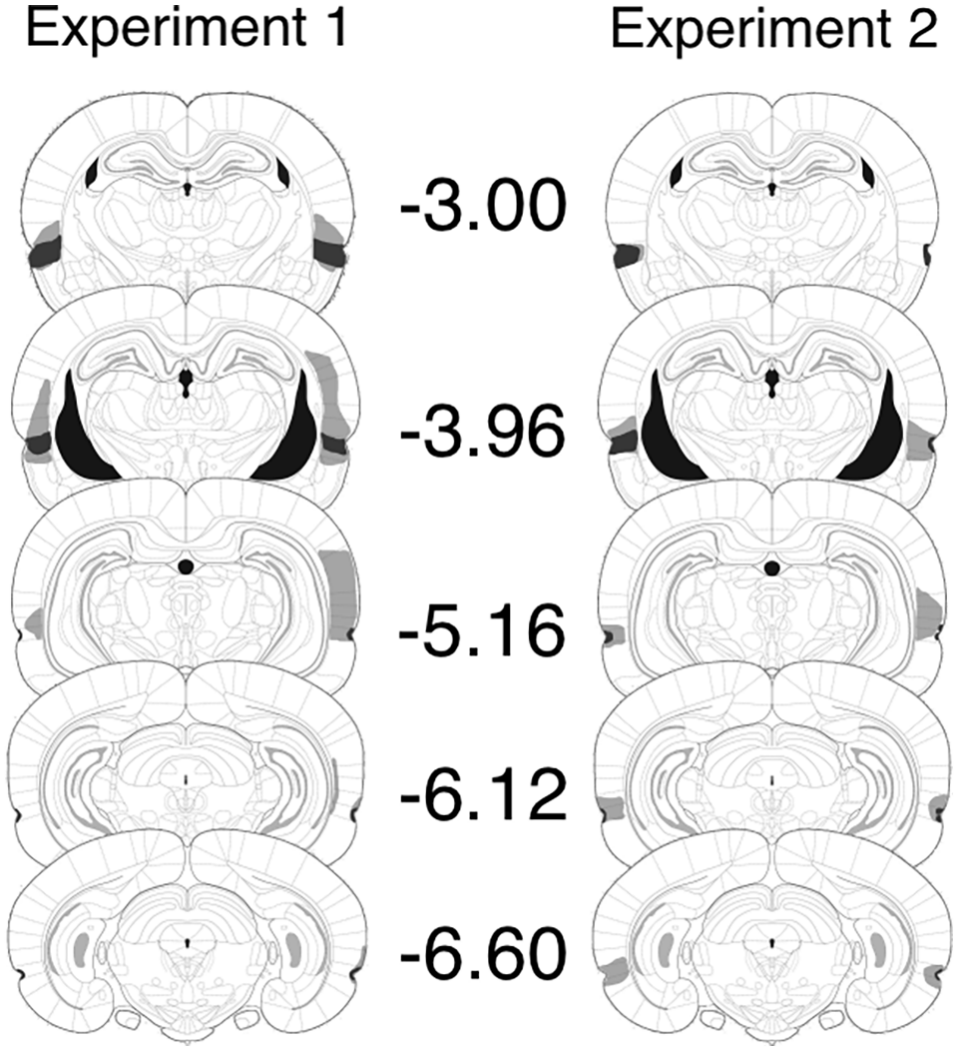
Histological reconstructions for Experiments 1 (left) and 2 (right). The maximum (grey) and minimum (black) cases of perirhinal lesions at coronal sections, taken through the rostro–caudal axis of the brain. Numbers adjacent to each section refer to the approximate distance (in mm) from bregma. Sections are based on those from *The Rat Brain in Stereotaxic Coordinates*, by G. Paxinos and C. Watson, San Diego, CA: Academic Press. Copyright 2005. Adapted with permission from Elsevier.

Tissue loss and damage in seven of the lesioned rats was asymmetrical and those rats were excluded from our analysis. Of the remainder (*n* = 9), all rats had localised, bilateral damage to the perirhinal cortex. The left panel of Figure 1 depicts the extent of tissue loss in the two cases with the largest and the smallest lesions. Damage occurred in all cases through the majority of the rostro–caudal axis of the perirhinal cortex (Burwell & Amaral, 1998). In all rats, damage began rostrally, adjacent to the agranular insular cortex and ended at around the beginning of the postrhinal cortex. In some of the cases, there was sparing of either the deep or superficial lamina at a particular rostro–caudal position, though this was the exception: In the majority of sections, damage was across both deep and superficial laminae. All rats had some extra-perirhinal cortex damage though no single area was bilaterally damaged in more than 22% of cases. Te_v_, the ventral temporal association areas (Burwell & Amaral, 1998), lying immediately dorsal to area 36, was damaged in five cases, but in only two was the damage bilateral. The lateral entorhinal cortex was damaged in six cases, but only in two was the damage bilateral. There was damage to the amygdala in five cases, but in only two was the damage bilateral. One case had unilateral damage to the CA2 field of the hippocampus.

#### Sensory preconditioning

**Figure 2.**
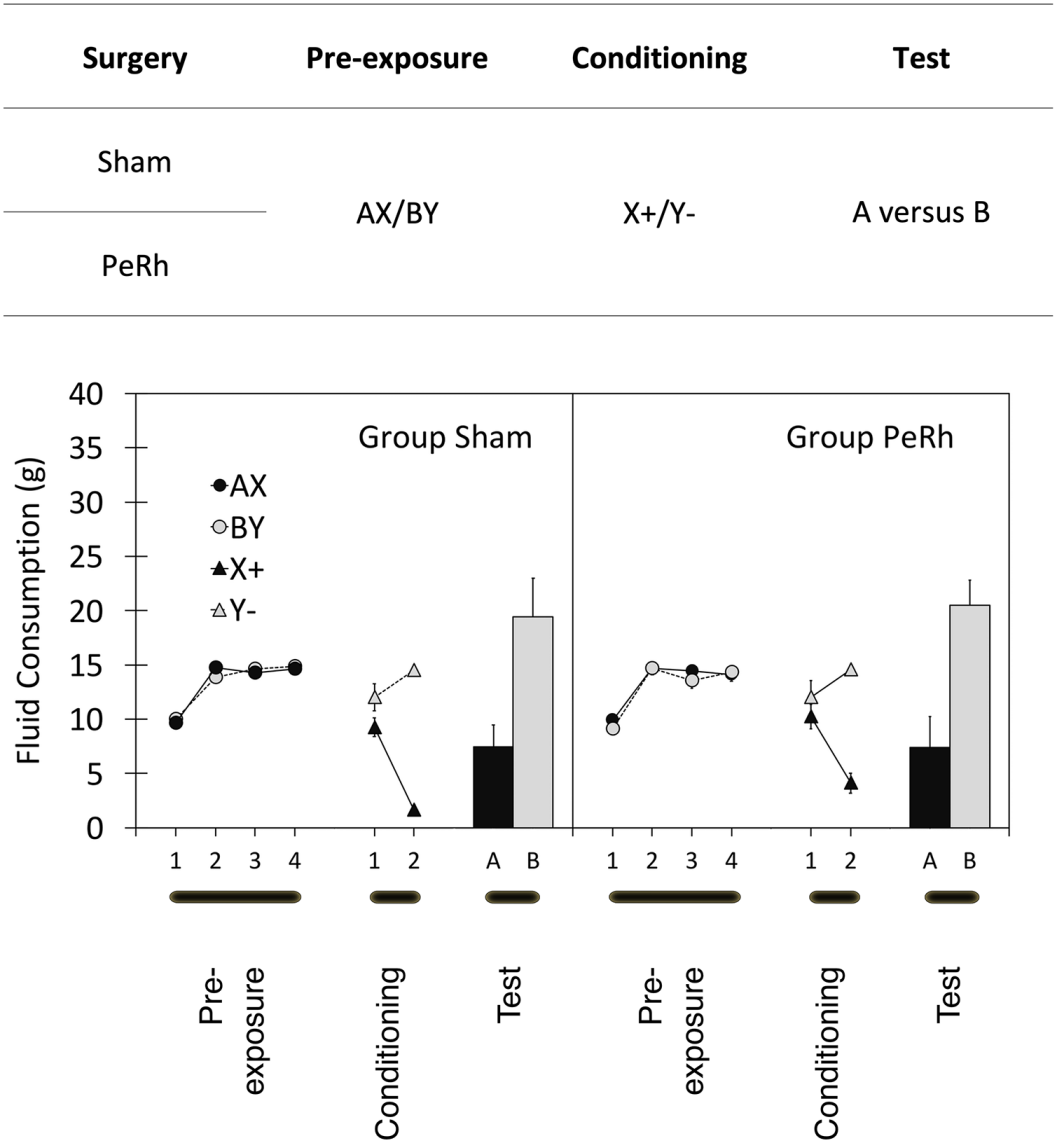
The design (top panel) and results (bottom panels) of the three stages of Experiment 1. A, B, X, and Y represent flavoured solutions and “+” and “−” the pairing or absence of, respectively, a lithium chloride injection. Adjacent letters (i.e., AX and BY) represent solutions of those two flavours. “/” indicates that two types of trial occurred in that stage of the experiment. Two groups of rats (group Sham and group PeRh) served in the experiment. Summary statistics represent mean amounts and standard deviations of consumptions of flavoured solutions in each of the three stages of the experiment with group Sham’s on the left panel and group PeRh’s on the right panel. See text for complete details.

Data from Experiment 1 are summarised in the lower panel of Figure 2. During pre-exposure, rats drank most of the cocktails that they were offered and consumption of AX and BY was similar, as was consumption across groups Sham and PeRh. That description was confirmed by analysis of variance (ANOVA). Only the main effect of trial was reliable, *F*(3, 45) = 114.2, *p* < .001, mean square error (MSE) = 191.0, $\eta_{\text{p}}^{\text{2}}=.884$ , 90% CI = [.817, .908], merely reflecting the increase in the amount of fluid offered (see “Procedure” section). The main effect of surgery was *F*(1, 15) = .2, *p* > .200, $\eta_{\text{p}}^{\text{2}}=.106$ . Trial’s interaction with surgery was *F*(1, 15) = 0.8, *p* > .494, $\eta_{\text{p}}^{\text{2}}=.051$ , and its interaction with flavour was *F*(3, 45) = 0.4, *p* > .727, $\eta_{\text{p}}^{\text{2}}=.028$ . The main effect of flavour was *F*(1, 15) = 0.4, *p* > .519, $\eta_{\text{p}}^{\text{2}}=.028$ . Its interaction with surgery was *F*(1, 15) = 0.6, *p* > .445, $\eta_{\text{p}}^{\text{2}}=.039$ and its interaction with trial was *F*(1, 15) = 0.4, *p* > .726, $\eta_{\text{p}}^{\text{2}}=.028$ . The triple interaction was *F*(3, 45) = 1.3, *p* > .262, $\eta_{\text{p}}^{\text{2}}=.084$ .

During conditioning, X was used to signal illness induced by lithium chloride injection, whereas Y was presented non-reinforced. Consumption of X and Y was similar on the first trial, but strong discrimination was demonstrated by Trial 2. ANOVA of these data confirmed that description revealing a main effect of trial, *F*(1, 15) = 7.9, *p* < .014, MSE = 79.8, $\eta_{\text{p}}^{\text{2}}=.347$ , 90% CI = [.048, .558], flavour, *F*(1, 15) = 119.7, *p* < .001, MSE = 821.1, $\eta_{\text{p}}^{\text{2}}=.889$ , 90% CI = [.753, .925], and a Trial × Flavour interaction, *F*(1, 15) = 46.6, *p* < .001, MSE = 373.9, $\eta_{\text{p}}^{\text{2}}=.757$ , 90% CI = [.502, .838]. The source of that interaction was examined using simple main effects analysis with the pooled error-term (SME). This revealed a lower consumption of X than Y on Trial 1, *F*(1, 16) = 5.4, *p* < .035, MSE = 43.4, $\eta_{\text{p}}^{\text{2}}=.253$ , 90% CI = [.012, .479], and on Trial 2, *F*(1, 16) = 143.6, *p* < .001, MSE = 1,151.6, $\eta_{\text{p}}^{\text{2}}=.889$ , 90% CI = [.783, .932]. No other statistic from the ANOVA was reliable. The main effect of surgery was *F*(1, 15) = 1.9, *p* > .187, $\eta_{\text{p}}^{\text{2}}=.113$ . The Flavour × Surgery and Trial × Surgery interactions were, respectively, *F*(1, 15) = 1.7, *p* > .200, $\eta_{\text{p}}^{\text{2}}=.106$ and *F*(1, 15) = 0.2, *p* > .616, $\eta_{\text{p}}^{\text{2}}=.017$ . The triple interaction was *F*(1, 15) = 0.281, *p* > .604, $\eta_{\text{p}}^{\text{2}}=.018$ . The X − Y difference on Trial 1 is not taken to be important and reflects only the fact that all rats received X+ as their first trial.

The data of central importance are those from the test of flavours A and B. Initial examination of the data revealed a general preference for consumption of sucrose relative to saline and, therefore, scores were simply adjusted to accommodate that bias (see, Robinson, 2017). There was no group difference in overall consumption of either sucrose, *t*(15) = .327, *p* > .747, or of saline, *t*(15) = .516, *p* > .613. Irrespective of its role as A or B, each rat’s sucrose score was multiplied by g/s, where g is the group mean flavour consumption of both sucrose and saline and s is the group mean consumption of sucrose alone. This yielded sucrose correction ratios of 0.71 and 0.78, respectively, for groups Sham and PeRh. The corresponding computation for saline yielded saline correction ratios of 1.71 and 1.38, respectively, for groups Sham and PeRh. These corrected data are summarised in Figure 2 and show consumption of flavour A to be reduced relative to that of B in both groups Sham and PeRh; furthermore, that bias was similar in the two groups. ANOVA confirmed that description in yielding only a main effect of flavour, *F*(1, 15) = 15.4, *p* < .002, MSE = 1,328.3, $\eta_{\text{p}}^{\text{2}}=.508$ , 90% CI = [.170, .672]. The surgery main effect was *F*(1, 15) = 0.0, *p* > .819, $\eta_{\text{p}}^{\text{2}}=.004$ , and its interaction with flavour was *F*(1, 15) = 0.0, *p* > .871, $\eta_{\text{p}}^{\text{2}}=.002$ . This bias in consumption constitutes demonstration of standard, gustatory sensory preconditioning (Rescorla & Cunningham, 1978; see also, Dwyer et al., 2012; Ward-Robinson et al., 2005). More important for our present concerns is the new finding that lesions of the perirhinal cortex influenced neither the presence nor the extent of sensory preconditioning.

The interpretation above rests on the ANOVA’s failure to find Surgery × A–B interactions, which is ambiguous: It could reflect genuine support for the position that the perirhinal cortex is unimportant for sensory preconditioning but it could also be the result of, for example, too low a sample size. A more direct source of evidence was sought by performing a Bayesian analysis. Its null model corresponding to the combined sensory preconditioning and surgery models (surgery + sensory preconditioning) was preferred over the model for the interaction of the two models ([surgery + sensory preconditioning] + [surgery × sensory preconditioning]) (382.048/147.369 = 2.592). This finding produces some evidence that the surgery variable did not influence the magnitude of sensory preconditioning. This is more direct evidence than that provided by the ANOVA’s failures to detect surgery × sensory preconditioning interactions, above.

## Experiment 2

**Figure 3.**
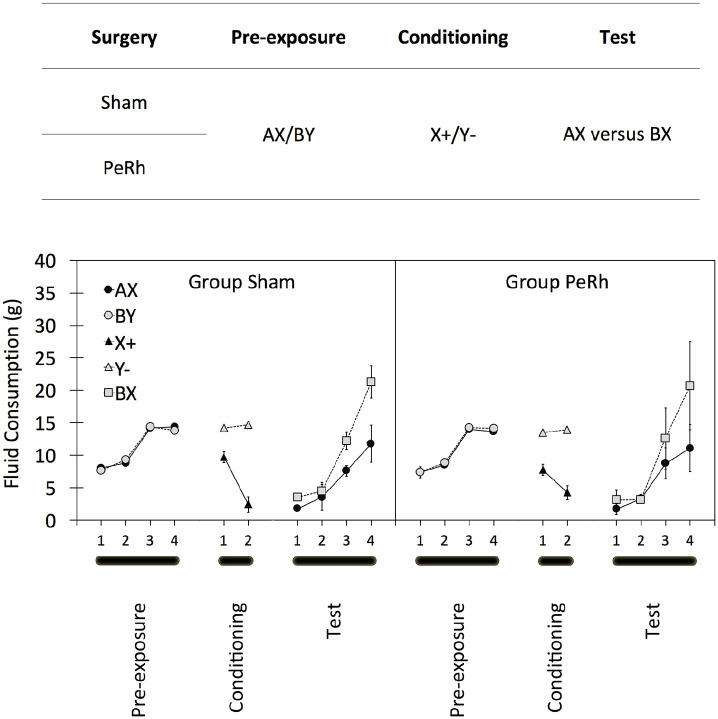
The design (top panel) and results (bottom panels) of the three stages of Experiment 2. A, B, X, and Y represent flavoured solutions and “+” and “−” the pairing or absence of, respectively, a lithium chloride injection. Adjacent letters (i.e., AX, BY, and BX) represent solutions of those two flavours. “/” indicates that two types of trial occurred in that stage of the experiment. Two groups of rats (group Sham and group PeRh) served in the experiment. Summary statistics represent mean amounts and standard deviations of consumptions of flavoured solutions in each of the three stages of the experiment with group Sham’s on the left panel and group PeRh’s on the right panel. See text for complete details.

Experiment 1 demonstrated that Rescorla and Cunningham’s (1978) sensory preconditioning procedure was not dependent on the perirhinal cortex: The sensory preconditioning effect was similar in group Sham and group PeRh. We discussed two mechanisms of test performance in sensory preconditioning: An associative chain of the form, A → X → + (cf. Dwyer & Killcross, 2006; Wagner, 1981; Ward-Robinson & Hall, 1998) and a form mediated by a unitary AX representation (cf. Rescorla, 1981). We suggested that this procedure, having simultaneously presented AX in pre-exposure, would encourage the latter, AX-mediated process, but Experiment 1 provided no direct evidence for it. Experiment 2 repeated Experiment 1 but included a modified test designed to demonstrate the possibility that the associative chain was an incomplete source of sensory preconditioning; its design is summarised in Figure 3.

Following Rescorla and Freberg (1978), we employed a test of sensory preconditioning in which the conditioned stimulus, X, was included: Consumption of AX and BX was compared, rather than the standard A–B comparison. This test does not eliminate any role of an associative chain, but it offsets any contribution that one could make in A’s consumption because both stimuli contain the actual X stimulus, rather than merely its associatively activated representation. However, an appreciable contribution of the AX-mediated form of learning in sensory preconditioning might lead to depression of AX relative to that of BX because of mediation by a unified AX representation. If the perirhinal cortex-dependent effects reported by Holmes et al. (2013) were the result on an effect on an A → X → + associative chain, and if A–B discrimination of Experiment 1 was based on a different AX-mediation process, we might, again, anticipate no influence of the perirhinal cortex lesion: that is, depression of AX relative to BX consumption in both surgical groups of rats.

### Method

#### Subjects and surgery

In total, 24 male rats of identical strain and similar background to those of Experiment 1 were used. Rats had served in two Skinner box experiments under food restriction. In one, rats responded on an instrumental baseline for food reinforcement and were presented with visual stimuli (the operation of two lamps, within the Skinner box). The other was a food-reinforced, Pavlovian discrimination that employed two auditory stimuli and one visual stimulus. Rats also served in arena experiments, as described for rats in Experiment 1. Surgery was performed in an identical manner to Experiment 1, 4 months before this procedure. Rats’ mean weight before the experiment began was 422 g (range: 375–470 g), and there was no weight difference between groups Sham and PeRh, *t* < 1.

#### Apparatus and stimuli

The apparatus and stimuli were identical to those of Experiment 1.

#### Procedure

The procedure followed a similar pattern to that of Experiment 1 except that (a) testing involved choice of the compound flavours AX and BX, rather than of the flavours A and B; (b) a series of four daily AX–BX test were given, rather than a single test. This was because the presence of directly reinforced X would, until the onset of extinction, suppress responding to the flavoured solutions, potentially masking any differences.

### Results and discussion

#### Histology

Tissue loss was asymmetrical in six lesioned rats and their data were eliminated. All remaining rats (*n* = 9) had localised, bilateral damage to the perirhinal cortex. The right panel of Figure 1 depicts the extent of the two cases of these remaining rats, having the largest and the smallest lesions. Lesions began at −3 mm from bregma and extended to −7 mm. There was some damage to dorsally adjacent areas, including ventral temporal association areas (five cases). There were two cases of damage to the ventral auditory area. The damage in these extra-perirhinal areas was unilateral.

#### Sensory preconditioning

Experiment 2’s data are summarised in the lower panel of Figure 3. As in Experiment 1, there appeared to be no differences during pre-exposure, either between groups Sham and PeRh or between AX and BY. And again, all rats’ consumption increased in line with the 5 mL increase in available fluid over the first and second pair of trials. ANOVA on these data supported that characterisation of the data, obtaining a main effect of trial only, *F*(3, 45) = 299.3, *p* > .001, MSE = 393.1, $\eta_{\text{p}}^{\text{2}}=.952$ , 90% CI = [.924, .962]. The main effect of surgery was *F*(1, 15) = 1.9, *p* > .184, $\eta_{\text{p}}^{\text{2}}=.114$ . Its interactions with flavour and trial were, respectively, *F*(1, 15) = 0.5, *p* > .446, $\eta_{\text{p}}^{\text{2}}=.036$ , and *F*(1, 15) = 0.1, *p* > .942, $\eta_{\text{p}}^{\text{2}}=.009$ . The Flavour × Trial interaction was *F*(1, 15) = 0.5, *p* > .673, $\eta_{\text{p}}^{\text{2}}=.033$ , and the triple interaction was *F*(1, 15) = 0.4, *p* > .702, $\eta_{\text{p}}^{\text{2}}=.310$ .

The X+/Y− given in the conditioning stage established a clear discrimination. ANOVA of these data yielded reliable main effects of flavour, *F*(1, 15) = 161.8, *p* < .001, $\eta_{\text{p}}^{\text{2}}=.915$ , 90% CI = [.809, .942], and trial, *F*(1, 15) = 26.1, *p* < .001, MSE = 103.1, $\eta_{\text{p}}^{\text{2}}=.635$ , 90% CI = [.317, .758], but the surgery main effect was unreliable, *F*(1, 15) = 0.5, *p* > .519, $\eta_{\text{p}}^{\text{2}}=.032$ . The Flavour × Trial interaction was reliable, *F*(1, 15) = 55.6, *p* < .001, MSE = 146.4, $\eta_{\text{p}}^{\text{2}}=.788$ , 90% CI = [.557, .858], but neither the Trial × Surgery, *F*(1, 15) = 3.7, *p* > .070, MSE = 15.0, $\eta_{\text{p}}^{\text{2}}=.202$ , nor the Flavour × Surgery interaction, *F*(1, 15) = 0.2, *p* > .613, MSE = 1.7, $\eta_{\text{p}}^{\text{2}}=.017$ , 90% CI = [.557, .858] was reliable. A reliable triple interaction was also obtained, *F*(1, 15) = 6.0, *p* < .028, MSE = 15.8, $\eta_{\text{p}}^{\text{2}}=.287$ , 90% CI = [.020, .511], whose source was examined with a pair of separate ANOVAs for each group.

The ANOVA on group Sham’s data revealed main effects of flavour, *F*(1, 7) = 89.9, *p* < .001, MSE = 556.9, $\eta_{\text{p}}^{\text{2}}=.928$ , 90% CI = [.730, .954], trial, *F*(1, 7) = 26.5, *p* < .002, MSE = 92.8, $\eta_{\text{p}}^{\text{2}}=.791$ , 90% CI = [.366, .871], and a Flavour × Trial interaction, *F*(1, 7) = 34.5, *p* < .001, MSE = 122.0, $\eta_{\text{p}}^{\text{2}}=.831$ , 90% CI = [.455, .895]. That interaction’s source was examined with SMEs. This revealed a lower consumption of X than Y on Trial 1, *F*(1, 7) = 22.2, *p* < .003, MSE = 78.77, $\eta_{\text{p}}^{\text{2}}=.760$ , 90% CI = [.308, .853] and on Trial 2, *F*(1, 7) = 169.8, *p* < .001, MSE = 600.2, $\eta_{\text{p}}^{\text{2}}=.960$ , 90% CI = [.844, .974].

The corresponding ANOVA for group PeRh revealed no main effect of trial, *F*(1, 8) = 4.8, *p* < .060, MSE = 21.0, $\eta_{\text{p}}^{\text{2}}=.377$ , 90% CI = [.000, .617], but a main effect of flavour, *F*(1, 8) = 74.0, *p* < .001, MSE = 532.8, $\eta_{\text{p}}^{\text{2}}=.902$ , 90% CI = [.682, .938], and a Flavour × Trial interaction, *F*(1, 8) = 19.0, *p* < .003, MSE = 35.0, $\eta_{\text{p}}^{\text{2}}=.704$ , 90% CI = [.258, .817]. Subsequent SMEs revealed consumption of X to be less than that of Y on Trial 1, *F*(1, 8) = 80.3, *p* < .001, MSE = 147.3, $\eta_{\text{p}}^{\text{2}}=.909$ , 90% CI = [.701, .942], and on Trial 2, *F*(1, 7) = 229.1, *p* < .001, MSE = 420.5, $\eta_{\text{p}}^{\text{2}}=.966$ , 90% CI = [.881, .978]. Thus, despite some indication that discrimination had been solved at different rates across the two groups, both groups successfully solved the discrimination.

The data of central importance are those of the test in which consumption of AX and BX was compared, and they are summarised in Figure 3. As in Experiment 1, data are corrected for preference of sucrose over saline. The new inclusion of separate correction ratios for the four test trials (in addition to those for the two groups and the sucrose and saline compounds, used in Experiment 1) resulted in 16 ratios being used. The four sucrose correction ratios, ranged from 0.51 to 0.60 for group Sham and 0.51 to 0.53 for group PeRh. The four saline ratios ranged from 3.07 to 20.35 for group Sham and from 9.75 to 47.67 for group PeRh. Thus, there was an indication that, irrespective of its role as AX or as BX, saline was consumed less than in Experiment 1 and that this may have been especially marked in group PeRh. However, there was no influence of surgery on the consumption of the sucrose or saline compounds: An ANOVA with group, test trial, and sucrose/saline compound revealed no main effect of surgery, *F*(1, 15) = 0.1, *p* > .712, MSE = 1.8, $\eta_{\text{p}}^{\text{2}}=.009$ , 90% CI = [.000, .178]. Main effects of trial, *F*(3, 45) = 193.5, *p* < .001, MSE = 1,368.5, $\eta_{\text{p}}^{\text{2}}=.928$ , 90% CI = [.886, .943], and sucrose/saline compound, *F*(1, 15) = 550.3, *p* < .001, MSE = 7,940.9, $\eta_{\text{p}}^{\text{2}}=.973$ , 90% CI = [.939, .982], were obtained. The Trial × Sucrose/saline compound interaction was reliable, *F*(3, 45) = 169.1, *p* < .001, MSE = 1,335.5, $\eta_{\text{p}}^{\text{2}}=.919$ , 90% CI = [.871, .935]. Neither the Trial × Surgery interaction, *F*(3, 45) = 0.6, *p* > .631, MSE = 4.1, $\eta_{\text{p}}^{\text{2}}=.037$ , 90% CI = [.000, .104], nor the Surgery × Sucrose/saline compound interaction was reliable, *F*(1, 15) = 0.1, *p* > .710, MSE = 4.1, $\eta_{\text{p}}^{\text{2}}=.037$ , 90% CI = [.000, .178]. The triple interaction was not reliable, *F*(3, 45) = 0.9, *p* > .439, MSE = 7.3, $\eta_{\text{p}}^{\text{2}}=.058$ , 90% CI = [.000, .142].

Test consumption for both AX and BX began rather low and at a similar level to consumption of X alone when measured on the final trial of conditioning. Consumption increased over the four trials comprising the test and revealed reduced consumption of AX relative to BX. Discrimination between AX and BX was evident in both groups and to a similar extent. ANOVA on test data yielded reliable main effects of trial, *F*(3, 45) = 22.7, *p* < .001, MSE = 1,366.6, $\eta_{\text{p}}^{\text{2}}=.603$ , 90% CI = [.008, .487], and flavour, *F*(1, 15) = 5.1, *p* < .038, MSE = 523.8, $\eta_{\text{p}}^{\text{2}}=.257$ , 90% CI = [.008, .487] only. The Test × Flavour interaction was close to standard levels of reliability, *F*(3, 45) = 2.7, .055 < *p* < .056, MSE = 139.9. The main effect of surgery was *F*(3, 45) = 0.0, *p* > .903, MSE = 1.8, $\eta_{\text{p}}^{\text{2}}=.001$ . Surgery’s interactions with trial and with flavour were, respectively, *F*(3, 45) = 0.1, *p* > .976, MSE = 4.1, $\eta_{\text{p}}^{\text{2}}=.005$ and *F*(1, 15) = 0.0, *p* > .879, MSE = 2.4, $\eta_{\text{p}}^{\text{2}}=.002$ . The triple interaction obtained *F*(3, 45) = 0.0, *p* > .997, MSE = 0.7, $\eta_{\text{p}}^{\text{2}}=.001$ .

That pattern of results is incompatible with an account of sensory preconditioning in which performance is driven solely by an associative chain because both compounds contain the mediating element, X. For our current interests, the main consequence of this finding is that it encourages the belief that the perirhinal cortex is unimportant for gustatory sensory preconditioning, when it is, at least in part, governed by a mechanism other than an associative chain.

We noted above that there was no overall influence of surgery on consumption of saline or of sucrose, independently of their roles as AX and BX, but that the correction ratio for saline in group PeRh was larger than that of group Sham. To examine the potential for any differential group influence, we repeated the AX–BX analysis but used the same suite of correction ratios for both groups (i.e., ratios were computed for each permutation of sucrose/saline × the four test trials but with group designation ignored). Average consumptions showed the same increases as in the original analyses over the four test trials, *F*(3, 45) = 33.0, *p* < .001, MSE = 1,407.5, $\eta_{\text{p}}^{\text{2}}=.687$ , 90% CI = [.528, .752]. Group Sham’s consumption was, on average, 3.6 g higher than group PeRh, *F*(1, 15) = 5.1, *p* < .04, MSE = 432.2, $\eta_{\text{p}}^{\text{2}}=.255$ , 90% CI = [.008, .486]. The main effect of flavour, *F*(1, 15) = 7.4, *p* < .017, MSE = 663.8, $\eta_{\text{p}}^{\text{2}}=.332$ , 90% CI = [.041, .546] and its interaction with test trial, *F*(3, 45) = 3.7, *p* < .019, MSE = 165.6, $\eta_{\text{p}}^{\text{2}}=.199$ , 90% CI = [.021, .322], were reliable. Most importantly here, none of the remaining interactions were reliable. Surgery’s interactions with trial and with flavour were, respectively, *F*(3, 45) = 1.7, *p* > .173, MSE = 73.7, $\eta_{\text{p}}^{\text{2}}=.103$ and *F*(1, 15) = 0.7, *p* > .402, MSE = 66.0, $\eta_{\text{p}}^{\text{2}}=.047$ . The triple interaction was *F*(3, 45) = 0.2, *p* > .976, MSE = 8.2, $\eta_{\text{p}}^{\text{2}}=.012$ . These unreliable interactions all involved the surgery variable. Thus, the finding that AX–BX discrimination was similar in both groups was not an artefact of the between-group difference in saline correction ratio in the original analysis.

As for Experiment 1, interpretation of Experiment 2’s data hinges on the interpretation of the failure of the ANOVA to detect reliable Surgery × Flavour and Surgery × Flavour × Trial interactions. To better specify this apparent feature of the data, we performed a Bayesian analysis on them. The two interaction models were compared to the models based on their constituent components alone. The null model for the double interaction was preferred over that of the double interaction by a factor of about 4 (i.e., 0.807/0.208 = 3.937). The null model for the triple interaction was preferred over that of the triple interaction by a factor of over a hundred (i.e., 2.223 × 10^9^/8.228 × 10^6^ = 271.025). Thus, the original ANOVA’s unreliable interactions involving the surgery and flavour variables appear to reflect a genuine feature of the test data, rather than being merely its failure to detect those interactions.

## General discussion

Holmes et al. (2013; see also, Holmes et al., 2018; Nicholson & Freeman, 2000) demonstrated that inactivation of the perirhinal cortex profoundly reduced rats’ sensory preconditioning. Such findings concur with evidence from anatomy (Burwell & Amaral, 1998) and from object recognition experiments in rodents (e.g., Albasser et al., 2009; Liu & Bilkey, 2001; Mumby & Pinel, 1994; Warburton et al., 2003; Winters et al., 2004). We noted that sensory preconditioning could be governed by an associative chain (A → X → +; for discussion, see, for example, Lin et al., 2017; Robinson et al., 2017) and/or by a form of stimulus generalisation, mediated by a unitary, undifferentiated representation of the AX compound stimulus (see, for example, Lin et al.; Rescorla, 1981). We reasoned that Holmes et al.’s use of serial compound, with no stimulus overlap, would favour the associative chain mechanism and that the simultaneous presentation would favour the AX-mediation mechanism. Our experiments examined sensory preconditioning in control rats and in rats with bilateral, excitotoxic lesions of the perirhinal cortex using simultaneously presented gustatory stimuli. We examined sensory preconditioning using a specialised test (AX versus BX) that controls for performance by any associative chain. This test confirmed an additional source of sensory preconditioning.

Although we have considered two accounts of sensory preconditioning so far, there is a third possibility: representation-mediated conditioning (e.g., Dwyer & Killcross, 2006; Ward-Robinson & Hall, 1998). According to this account, A’s capacity to suppress consumption on test is the result of it gaining its own direct association with the effects of the lithium chloride. The pre-exposure to the AX compound supports the formation of excitatory associations (A → X and X → A). During subsequent X+ trials, the X → A association activates A’s representation, allowing it to enter into association with lithium chloride’s effects *directly* and supporting future test responding to A. Like the undifferentiated AX-mediation account (e.g., Rescorla, 1981), the representation-mediated conditioning account anticipates the results of the AX–BX test of Experiment 2, but for a different reason: A’s capacity to elicit the conditioned response is direct and independent of X’s association with the effects of lithium chloride. Thus, it is important to note that the AX–BX results in Experiment 2 violate the associative chain account, but they do not discriminate between accounts based on the representation-mediated conditioning of the undifferentiated AX account.

However, gustatory sensory preconditioning is seen when the conditioning stage (X+) is replaced by a *motivational* change in X’s value (e.g., Rescorla & Freberg, 1978; Westbrook et al., 1995). This feature of the procedure makes it impossible for the representation-mediated conditioning account to be generally applied to gustatory sensory preconditioning. The procedure involves saline serving as X with testing of A occurring under a state of pharmacologically induced salt appetite. Here, sensory preconditioning is demonstrated by an *increase*, rather than a reduction in A consumption. Evidence for representation-mediated learning as a mechanism of sensory preconditioning exists (e.g., Ward-Robinson & Hall, 1998), but salt-appetite demonstrations of sensory preconditioning imply that it plays no major role in gustatory sensory preconditioning. Even if we were to dismiss this evidence against the representation-mediated conditioning account of the current demonstration of gustatory sensory precondition, this does not detract from the fact that the AX–BX test results from Experiment 2 appear to show that the associative chain mechanism is unimportant irrespective of the integrity of the perirhinal cortex.

We acknowledge that during Experiment 2’s testing, BX’s novelty relative to AX could have induced a neophobic reaction, suppressing its consumption and causing a diminution of sensory preconditioning. Morillas et al. (2017) reported that rats with perirhinal cortex lesions consumed less acid-flavoured solution than their sham-lesioned comparison group during a general increase in daily consumption. They interpreted this as a showing that the perirhinal cortex was important for maximally effective habituation of neophobia, drawing a parallel with effects in recognition memory experiments (see also, Jones et al., 2012; Robinson et al., 2009). Any such effect here would enhance, rather than attenuate, group PeRh’s AX–BX test discrimination in Experiment 2. However, we saw no evidence for Morillas et al.’s lesion-induced attenuation of neophobia, either in the AX–BX test or during pre-exposure where such differences might be more likely to exhibit themselves.

Rescorla’s (1981) analysis of the AX-mediation account is not that A and X become associated or that there is any learning process about their co-occurrence—it is that they are undifferentiated. Differentiation can occur when A and X are presented separately, during extinction (e.g., Bailey & Westbrook, 2007; Rescorla & Freberg, 1978; Ward-Robinson et al., 1998) or in sensory preconditioning experiments, such as Holmes et al.’s (2013), which presented A → X pre-exposure trials serially. Nicholson and Freeman’s (2000) experiment also found a role for the perirhinal cortex in sensory preconditioning. Their procedure was similar to Holmes et al.’s in that serially presented audio–visual compounds were pre-exposed, and a shock reinforcer was used during conditioning. (Note—the temporal arrangements of Nicholson and Freeman’s pre-exposure is unspecified, but authors confirmed to us that the pre-exposure pairings were serial, not simultaneous; J Freeman, 13 August 2019, personal communication). From this position, there is no conflict in our and Holmes et al.’s and Nicholson and Freeman’s findings: The perirhinal cortex is involved in serial but not simultaneous sensory preconditioning. The implication of the discussion here is that the perirhinal cortex is involved in standard associative chain learning and/or performance but is not important for the AX-mediation source of sensory preconditioning. In addition to the serial/simultaneous presentation of A and X, many other differences exist between our procedures, including the means of means of modulating perirhinal cortex function and modalities of the stimuli used. To make this point decisively, experiments would need to demonstrate, within a single experiment, that perirhinal inactivation had different effects on AX-mediation and associative chain sources of sensory preconditioning.

It is important to recognise that brain lesions, in being permanent, may operate during any or all three stages of sensory preconditioning. Thus, had we found the perirhinal cortex lesions to be effective, we would be unable to identify it as a limitation in the learning during pre-exposure and/or conditioning or one on performance (i.e., a failure to access intact learning or translate that learning into the conditioned response). This problem is avoided by Holmes et al.’s (2013; see also Holmes et al., 2018) use of intracranial infusion. They found perirhinal cortex inactivation immediately after pre-exposure attenuated sensory preconditioning. Because the perirhinal cortex would have recovered by the subsequent conditioning and test stages of the procedure, this finding naturally points to the perirhinal cortex’s importance in learning about the A and X serial pairing during pre-exposure.

Another possibility for our failure to detect a role for the perirhinal cortex in sensory preconditioning is that the lesions were ineffective. However, the rats from Experiment 1 of the current study had served in a study that demonstrated a pronounced reduction in novelty/familiarity generalisation (Robinson et al., 2010). The surgery given to the rats of Experiment 2 of the current study was performed in an identical manner to Experiment 1’s, by the same surgeon (P.M.J.) using the same surgery. Furthermore, our analysis of histology, revealed suitably sized and positioned perirhinal cortex lesions in all retained rats. However—as with all null lesion effects—it is possible that the perirhinal cortex is importantly involved in gustatory sensory preconditioning but that a different brain system takes responsibility for this in reaction to the perirhinal cortex lesion. Such a compensation process could mask a genuine, albeit temporary, deficit. It is also notable that our procedures were performed a relatively long period after surgery, raising the possibility of some functional recovery. Another advantage of the reversible lesions considered above (e.g., Holmes et al., 2013) is that brain inactivation is too brief to realistically encourage alternative brain systems to compensate. Thus, any failure to detect a temporary lesion’s effect is less amenable to any explanation based on compensation by alternative brain systems.

Three additional temporal lobe brain regions have also been found to be unimportant using the gustatory sensory preconditioning procedure. Ward-Robinson et al. (2001) and Ward-Robinson et al. (2005) gave rats bilateral, excitotoxic lesion of, respectively, the hippocampus and the adjacent entorhinal cortex, which are both connected to the perirhinal cortex. Lesioned rats’ performance was indistinguishable from rats that had received only sham surgery. Blundell et al. (2013) and Dwyer and Killcross (2006) reported that rats with bilateral, excitotoxic lesions of the basolateral amygdala were unaffected in their gustatory sensory precondition; however, each report describes deficits in other related tasks. Blundell, et al. found that basolateral amygdala lesions reduced flavour-potentiated odour aversion learning. Two groups of sham-lesioned rats drank either water or saline, infused with an odorant; that is, the solutions, respectively, had only an odour or both an odour and a flavour. Drinking was followed by an injection of lithium chloride. Both groups of rats were subsequently tested for a lithium-induced aversion to the odour alone. The addition of the flavour to the odour during conditioning resulted in an aversion to the odour. These two treatments were also given to amygdala-lesioned rats. Their standard flavour-potentiated odour aversion was reduced, demonstrating a role for the basolateral amygdala. This finding is intriguing because flavour-potentiated odour aversions (e.g., Droungas & LoLordo, 1991) have been demonstrated to rely on the same processes as sensory preconditioning (Rescorla & Durlach, 1981). Dwyer and Killcross found reduced sensory preconditioning in rats with lesions of the basolateral amygdala when it relied on pre-exposure to a flavoured solution in a distinct context (a particular arm of a Y maze). After pre-exposure, rats received conditioning in which they received a lithium chloride injection in that Y-maze arm. Consumption of the pre-exposed flavour was assessed in rats’ home cages and compared to consumption of a control flavour. Control rats’ consumed less of the pre-exposed flavour than the control flavour—the standard sensory preconditioning results—but this discrimination was reduced in lesioned rats. Thus, in both Blundell et al.’s and in Dwyer and Killcross’ reports, the switch of the conditioned stimulus, X, from a flavour to, respectively, an odour or a context reveals a sensitivity to basolateral amygdala lesions. Dwyer and Killcross suggested that the dissociation was based on two distinct mechanisms underlying the two sensory preconditioning procedures: an associative chain, when X was a flavour, and an A-mediated conditioning process, when X was a context. According to this account, stimulus A gains its own, direct, associative strength, when it is associatively activated by X during conditioning and its representation is paired with and becomes associated with the reinforcer—in this case, the effects of the lithium chloride. Although there was no direct evidence for A-mediated conditioning—as opposed to any other possible mechanism—this seems a reasonable explanation of their results and could apply to Blundell et al.’s results too. An alternative explanation is that the basolateral amygdala is important for learning about the co-occurrence of bimodal (i.e., odour and flavour; maze and flavour), but not unimodal elements. However, this suggestion appears to be incorrect because another aspect of Holmes et al.’s (2013) study involved amygdala inactivation with their bimodal (audio–visual) procedure but this did not affect sensory preconditioning. Despite showing evidence of aberrant sensory-specific learning in another task, Scarlet et al. (2012) found no influence of basolateral amygdala lesions on rats’ performance in a task that is structurally identical to a gustatory sensory preconditioning experiment. However, they point out that, in the concentrations that they used their flavoured solutions, two of the elements (sucrose and polycose) were sufficiently concentrated to act as a nutritional stimulus. Thus, they may have been less neutral than in standard sensory preconditioning procedures making comparison with the other experiments less clear.

One objection to our acceptance of alternatives to the associative chain account could be made based on potentially different levels of processing of X during the AX–BX test. The conditioned response to X might be reduced by the addition of either A or B on the AX–BX test, increasing consumption. Furthermore, perhaps BX’s relative novelty could cause relatively great interference of X’s processing, elevating consumption relative to that of AX, familiar from pre-exposure. Notice that an associative chain account could now anticipate that BX consumption should exceed AX consumption. From here, we need to assume that the suppression caused by BX’s relative novelty is a less powerful influence on test consumption than its interference with processing of X. This argument remains possible but seems weakened by its reliance on learning about specific combinations of A, B, and X, which can be taken to be the form of learning underpinning the configural learning mechanism described above. Thus, the enhanced responding of BX relative to AX probably does indicate that any role for an associative chain can be at best an incomplete account.
